# Family Caregiving III A Telephone & Online Intervention for Spanish-Speaking Post-Stroke Caregivers: Enhancing Self-Efficacy

**DOI:** 10.1093/geroni/igaf122.2970

**Published:** 2025-12-31

**Authors:** Janet Lopez, Melanie Orejuela, Nathaniel Eliazar-Macke, Arkaprava Roy, Tatiana Orozco, I Freytes

**Affiliations:** University of Central Florida, Orlando, Florida, United States; US Department of Veterans Affairs, Gainesville, Florida, United States; Veterans Health Administration, Miami, Florida, United States; University of Florida, Gainesville, Florida, United States; US Department of Veterans Affairs, Gainesville, Florida, United States; US Department of Veterans Affairs, Gainesville, Florida, United States

## Abstract

Caregivers play a crucial role in managing the health and well-being of Veterans with stroke. However, the demands of caregiving can significantly impact their self-efficacy, which is essential for effective caregiving. While there is some evidence suggesting interventions can improve self-efficacy among stroke caregivers, little is known about self-efficacy among Spanish speaking caregivers of Veterans. We report the impact of a problem-solving telephone and online intervention on Spanish-speaking caregivers of Veterans post-stroke’s self-efficacy. The study was a two-arm parallel randomized controlled trial with repeated measures and mixed methods that tested the effect of a telephone and online problem-solving intervention for Spanish-speaking stroke caregivers. We utilized the Caregiver Self-Efficacy tool (Spanish Version), a 15-item measure assessing caregivers’ confidence in obtaining respite, responding to disruptive patient behaviors, and controlling upsetting thoughts. Self-efficacy was assessed at baseline, post-intervention 1, and post-intervention 2 in 105 participants. Wilcoxon rank sum test for statistical analysis. At baseline, there were no significant differences between the intervention and standard care groups across all domains. Post-intervention 1 showed slight improvements in the intervention group, though not statistically significant. By post-intervention 2 (12 weeks), significant improvements were observed in the intervention group across all domains: obtaining respite care (p = 0.003), responding to disruptive behaviors (p = 0.018), and controlling thoughts (p = 0.045). The intervention significantly enhanced caregiver self-efficacy over time, particularly post intervention. Findings suggest that structured support and training can effectively boost Spanish-speaking caregivers’ confidence in managing caregiving tasks, ultimately improving Veterans and caregiver well-being.

